# Who is seeking antiretroviral treatment for HIV now? Characteristics of patients presenting in Kenya and South Africa in 2017‐2018

**DOI:** 10.1002/jia2.25358

**Published:** 2019-09-13

**Authors:** Alana T Brennan, Mhairi Maskew, Bruce A Larson, Isaac Tsikhutsu, Margaret Bii, Lungisile Vezi, Matthew P Fox, Willem DF Venter, Peter Ehrenkranz, Sydney Rosen

**Affiliations:** ^1^ Department of Global Health Boston University School of Public Health Boston MA USA; ^2^ Department of Epidemiology Boston University School of Public Health Boston MA USA; ^3^ Health Economics and Epidemiology Research Office Department of Internal Medicine School of Clinical Medicine Faculty of Health Sciences University of the Witwatersrand Johannesburg South Africa; ^4^ Kenya Medical Research Institute/U.S. Army Medical Research Directorate‐Africa Nairobi Kenya; ^5^ Henry Jackson Foundation MRI Kericho Kenya; ^6^ Wits Reproductive Health and HIV Institute Department of Internal Medicine School of Clinical Medicine Faculty of Health Sciences University of the Witwatersrand Johannesburg South Africa; ^7^ Bill and Melinda Gates Foundation Seattle WA USA

**Keywords:** HIV/AIDS, presenting for care, patient characteristics, randomized trial, SLATE I Kenya, SLATE II South Africa

## Abstract

**Introduction:**

Many African countries have had at least two years’ experience with universal treatment eligibility for HIV. The literature contains few descriptions, though, of populations starting treatment since adoption of universal eligibility. Using baseline data from a clinical trial of same‐day ART initiation, we describe the populations presenting for HIV testing or care at study clinics in Kenya and South Africa in 2017‐18, during the era of same‐day initiation.

**Methods:**

The Simplified Algorithm for Treatment Eligibility (SLATE) trials in Kenya (SLATE I) and South Africa (SLATE II) were multicenter, non‐blinded, individually randomized, pragmatic trials evaluating simple, standardized algorithms to determine eligibility for same‐day initiation of ART without relying on laboratory results, point of care tests or multiple clinic visits. In Kenya, enrolment occurred during July 2017 to April 2018. In South Africa, enrolment occurred during March to September 2018. We describe demographic, socioeconomic and clinical characteristics of patients randomized to the same‐day initiation arm for both studies.

**Results and Discussion:**

A total of 240 and 296 participants were enrolled in Kenya and South Africa. The majority were female (59% and 64% respectively), with a median age of 35 years. In both countries, most subjects were newly diagnosed with HIV on the day of enrolment (62%, 55%), believed they already had adequate knowledge to begin ART (78%, 68%), and preferred to start ART immediately (same‐day) (98% in both countries). About 40% of all patients had at least one symptom related to tuberculosis (cough, fever, night sweats, weight loss) and/or cryptococcal meningitis (continuous headache). More than a third of patients (37%, 36%) presented with advanced disease (CD4 <200 cells/mm^3^), a fifth presented with very advanced disease (CD4 < 100), and approximately 1 in 20 presented with very advanced disease and were asymptomatic.

**Conclusions:**

Despite >2 years of universal eligibility for ART in Kenya and South Africa, in 2017‐2018 more than half of HIV‐positive patients presenting at public sector clinics were not yet aware of their status, and more than a third presented for care with advanced HIV disease. These proportions remain similar to those observed before the introduction of universal eligibility.

## 1 Introduction

The World Health Organization's (WHO) 2016 consolidated guidelines recommended that all adults living with HIV should initiate antiretroviral treatment (ART) regardless of WHO clinical stage or CD4 cell count 1. This recommendation, known as Universal Testing and Treatment (UTT), was widely adopted in sub‐Saharan Africa over the course of the following year (see, for example, 2).

The expectation for UTT was that HIV‐positive individuals would present for ART initiation earlier than in the past, when eligibility for treatment required having a CD4 count below a specific threshold or an AIDS‐defining condition 3. Despite many African countries now having at least two years of experience with universal treatment eligibility for HIV, there are as of yet few published descriptions of populations presenting for treatment initiation since treatment eligibility became universal.

Recently collected baseline data for the Simplified Algorithm for Treatment Eligibility (SLATE I and SLATE II) trials in South Africa and Kenya, which enrolled patients presenting for routine HIV care but not on treatment, provide contemporary information on who is presenting for treatment initiation. Using baseline data from these companion clinical trials, we describe characteristics of patients presenting for HIV testing or care, but not yet on ART, from mid‐2017 to mid‐2018 in South Africa and Kenya. These data help to assess the early impact of universal treatment in promoting treatment uptake prior to disease progression.

## 2 Methods

The Simplified Algorithm for Treatment Eligibility (SLATE) trials in South Africa and Kenya are multicenter, non‐blinded, individually randomized (1:1), pragmatic trials evaluating simple, standardized algorithms to determine eligibility for same‐day initiation of ART without relying on laboratory results, point of care tests or multiple clinic visits 4, 5. The initial SLATE trial in both countries, called SLATE I (NCT02891135), was designed to address the concerns of clinicians and programme managers in 2015, when the algorithm was first proposed 6. Enrolment for SLATE I was completed in July 2017 in South Africa and April 2018 in Kenya. Using baseline data from SLATE I in South Africa, a revised algorithm incorporating an alternative approach to screening for tuberculosis and fewer reasons for delaying ART initiation was developed (SLATE II; NCT03315013) and evaluated in South Africa. Enrolment for STATE II was completed in September 2018.

The SLATE studies were carried out in typical public sector clinics in both countries. In Kenya, these were outpatient HIV clinics in county‐level hospitals in Kericho, Nandi and Kisumu counties. In South Africa, study sites were primary health clinics serving urban informal and formal communities in the Johannesburg and Ekurhuleni metropolitan areas. Patient inclusion and exclusion criteria for study eligibility are detailed in published protocols 4, 5. Those enrolled were non‐pregnant, HIV‐infected adults (18+ years) not currently on ART and willing to provide written informed consent. At the time of study enrolment, some patients had only learned of their HIV infection on that day, while others had already completed one or more “pre‐ART” visits leading up to treatment initiation.

In Kenya (SLATE I), the study was powered to detect a 15% difference in the primary outcome of initiation of ART within 28 days and alive, in care and retained on ART 8 months after study enrolment. We aimed for a sample size of 240 per arm (480 total) in Kenya and 300 per arm (600 total) in South Africa 4, 5.

SLATE I and II included a baseline questionnaire eliciting basic demographic and other information about prior HIV care. Patients in the intervention arms were assessed for eligibility for same‐day ART initiation by a study clinician using the SLATE I (Kenya) or SLATE II (South Africa) algorithm 4, 5. The algorithm consisted of four screens: (1) symptoms, (2) recent medical history, (3) physical conditions and (4) treatment readiness. We describe the characteristics and condition of intervention arm patients based on data obtained through the baseline questionnaire and screening instruments. Percentages and medians with interquartile ranges for key variables for each study are provided. Because parallel data on patient condition and readiness are not available for the standard of care arms, we report here only on patients randomized to the intervention arms.

## 3 Results and Discussion

### 3.1 Enrolment

In Kenya, 240 patients were randomized to the intervention arm in SLATE I between July 13, 2017 and April 17, 2018. In South Africa, 296 patients were randomized to the intervention arm in SLATE II between March 14 and September 18, 2018.

### 3.2 Demographic characteristics

Table 1 reports the demographic characteristics of patients enrolled and randomized to the intervention arm, stratified by country. In both countries, most participants (roughly 60%) were female and in their mid‐30s. Most other characteristics differed substantially between countries, however, reflecting the populations served by the study clinics. In Kenya, most patients resided in a rural area or village, lived in their primary home, and were employed mainly in the informal sector (for example, agricultural work, day labour and so on.), with only 5% reporting being unemployed and looking for work. In South Africa, most patients resided in a peri‐urban area in a non‐primary residence, and 47% reported being unemployed and seeking work. In Kenya, most were married and living in households with a median of three other household members. In South Africa, most were single and living with just one other household member. Most Kenyan patients used a taxi/minibus and/or motorbike as transport to their clinic, while the majority in South Africa walked. While a substantially larger proportion of patients in Kenya than South Africa incurred travel costs, the amounts paid by those who did were very similar.

**Table 1:** Characteristics of intervention arm patients in Kenya and South Africa

Variables^a^	Categories	Intervention arm		Intervention arm	
		Kenya (SLATE I)		South Africa (SLATE II)	
		(N = 240)		(N = 296)	
Period of enrolment		July 13, 2017‐April 27, 2018		March 14‐Sept 18, 2018	
		%	Number	%	Number
Sex	Female	59%	142	64%	189
Age	Median (IQR)	36 (29, 44)		35 (29, 41)	
Location of current residence	Town (urban)	15%	35	10%	31
	Peri‐urban	28%	66	90%	265
	Rural home or village	58%	139	0%	0
Marital status	Single	20%	49	58%	172
	Married or long‐term partner	49%	118	32%	94
	Divorced, separated, or widowed	30%	73	10%	30
Current house is primary residence	Yes	64%	154	43%	128
Number other persons in house	Median (IQR)	3 (2, 5)		1 (1, 3)	
Usual activity when well^b^	Formal employment	8%	18	23%	69
	Informal sector work	67%	161	24%	70
	Unemployed, looking for work	5%	12	47%	138
	Unpaid domestic work	15%	36	1%	3
	Studying or training	1%	3	1%	4
	Other	4%	9	4%	12
Transport mode to clinic today (multiple choices possible)	Minibus or taxi	49%	118	37%	110
	Walking	19%	46	54%	161
	Private car	5%	11	8%	25
	Motorbike	57%	137	0%	0
Paid to travel to clinic today	Yes	86%	206	46%	136
Travel cost one way, if any cost	Median (IQR)	$0.69 (0.49‐0.98)		$0.73 (0.57‐0.89)	
Travel time one way (min)	Median (IQR)	30 (20, 50)		15 (10, 30)	

a Percentages are rounded to the nearest whole number. Travel costs are reported in 2018 US dollars based on 101.8 KES/$ and 12.3 ZAR/$ (the average exchange rates for 2018, January to June 2018 reported in the International Monetary Fund Financial Statistics 7

b For the variable “usual activity when well,” one observation is missing for Kenya.

### 3.3 Patient HIV experience and preferences

The majority in both countries had not visited the study clinic for any kind of HIV care prior to the baseline study visit (Table 2). Common reasons for making a clinic visit on the day of study enrolment included (1) having an HIV test; (2) any pre‐ART care or monitoring, including preparation to start ART; and/or (3) a clinical visit because they were unwell. Most patients had not tested positive for HIV prior to the visit on the day of study enrolment, but most reported having had an HIV test, presumably negative, in the past. Most patients reported having adequate information to decide if and when they wanted to start ART, and essentially all patients reported wanting to start ART that day.

**Table 2:** Patient HIV‐care experience and preferences for initiation^a^

Variable	Categories	SLATE Arm		SLATE II Arm	
		Kenya		South Africa	
		(N = 240)		(N = 296)	
		%	Number	%	Number
Previously visited this clinic for any kind of HIV care	No	81%	195	68%	202
	Yes	19%	45	32%	94
Reason for today's visit (multiple choices possible)	HIV test (diagnosis)	48%	114	36%	108
	HIV care or monitoring	34%	81	48%	141
	Get care because did not feel well	29%	69	12%	36
	Reason not related to HIV care/treatment	2%	5	4%	11
Ever tested positive before today	No	62%	148	55%	163
	Yes	38%	92	45%	133
Year first tested positive for HIV (if tested positive before today)	N tested positive before today	92		133	
	Prior to 2010	10%	9	6%	8
	2010‐2013	21%	19	15%	20
	2014‐2016	9%	8	23%	31
	2017‐2018	61%	56	56%	74
Expected to start ART today	No	47%	113	38%	113
	Yes	53%	127	62%	183
If not expecting to start today, reason not expecting to start today (multiple reasons possible)	N not expecting to start		113		113
	First visit to site	6%	7	21%	24
	HIV status unknown before today	86%	97	49%	55
	Not eligible for treatment yet	1%	1	4%	4
	More visits expected	2%	2	25%	28
	Not told I was going to start	3%	3	8%	9
	I do not want to receive medications today	0%	0	0%	0
	Other reason	5%	6	4%	5
Disclosed status to anyone else (if tested positive before today)	N tested positive before today		92		133
	No	23%	21	17%	22
	Yes	77%	71	83%	111
Do you have enough information to start ART today?	I already know enough	78%	186	68%	201
	I would like a little more information	17%	40	22%	65
	I would like a lot more information	6%	14	10%	30
When would you prefer to start ART?	Today	98%	235	98%	289
	Within next week (but not today)	1%	3	2%	6
	Within next month (but not next week)	0%	1	0%	1
	Sometime in next 6 months (but not this month)	0%	0	Not asked	
	I am not ready to start	0%	1	0%	0

a The percentages in Table 2 are rounded to the nearest whole number.

### 3.4 Symptoms

In Table 3 we present the prevalence of five symptoms self‐reported at the study enrolment visit: cough, fever, night sweats, weight loss and continuous headache. The first four comprise the standard TB screen; the last is a common symptom of cryptococcal meningitis. In addition, patients were asked about any other symptoms that might warrant further clinical assessment or investigation. About 40% of all patients had one or more of these symptoms, with cough and weight loss being most common in both countries, and fever, night sweats and headache more common in Kenya than in South Africa. Most patients who reported any symptoms had multiple symptoms.

**Table 3:** Self‐reported symptoms, medical history, physical examination findings and readiness assessment

Variable (% responding yes)^a^	Intervention arm		Intervention arm	
	Kenya (SLATE I)		South Africa (SLATE II)	
	(N = 240)		(N = 296)	
	%	Number	%	Number
Symptom presence (self‐reported)				
Cough (current)	33%	78	26%	76
Fever	23%	54	7%	20
Night sweats	24%	58	10%	29
Weight loss	31%	75	32%	96
Headache	13%	31	4%	12
Number of symptoms (self‐reported)				
0 symptoms	60%	144	53%	156
Any symptom (1 or more of the above)	40%	96	47%	140
1 symptom	5%	11	27%	80
2 symptoms	9%	21	12%	36
3 or more symptoms	27%	64	8%	24
Medical history				
On ART before (previous defaulter)	8%	18	11%	33
On TB treatment now	1%	3	0%	0
Other condition	3%	6	1%	3
Taking epilepsy medication	0%	0	Not asked	
Taking Warfarin	0%	0	Not asked	
Taking any other medications suggesting further consultation?	1%	3	1%	4
Brief physical examination				
Underweight (BMI<18.5)	21%	51	7%	20
Elevated blood pressure (systolic > 140 or diastolic > 90)	9%	22	24%	72
Other condition	12%	29	2%	6
Any exam finding that could affect ART initiation	3%	7	4%	11
Readiness assessment				
Ready to start ART today	99%	237	98%	289

a Percentages are rounded to the nearest whole number. For the percentages in Table 3, n = 240 for Kenya and n = 296 for South Africa for all questions except as noted in here. Underweight (n = 223 in Kenya) is defined as body mass index < 18.5 8. Elevated blood pressure is defined as (systolic > 140 or diastolic > 90) 8, 9.

### 3.5 Medical history and physical examination findings

A brief medical history addressed topics relevant to ART initiation, including: (1) prior experience on ART; (2) TB treatment for less than two weeks; and (3) currently taking other medications that could complicate ART. Roughly one of the ten patients were reengaging in HIV care and treatment after initiating previously. Few patients had started recent TB therapy, and little pertinent information pertaining to ART initiation were identified in the medical histories.

Results of the physical examination revealed that few patients had a fever on the day of assessment, based on thermometer measurement by study clinician (although 23% of patients reported a current or recent history of fever in the symptoms screen in Kenya). A small share of patients in South Africa (7%) and a larger share in Kenya (22%) were underweight. Elevated blood pressure was common.

Following the physical exam, the clinician could recommend further assessment prior to ART initiation due to clinical signs/symptoms (noted as “exam finding” in Table 3) or conditions not reported in the self‐reported symptom screen that suggested further consultation. For example, a patient could report no symptoms of pulmonary TB, but have could have other signs or symptoms indicative of extrapulmonary TB (that is, palpable lymph nodes, pleuritis or swollen joints). Positive findings on clinical examination were uncommon in South Africa (6% of intervention arm patients) but were observed more frequently in Kenya (15%).

### 3.6 Readiness assessment

A simple readiness assessment was used to evaluate if a patient considered himself or herself ready to initiate ART. Nearly all patients confirmed they would prefer to start ART today if they could (that is, if offered the chance). This information from the readiness assessment with the clinician is consistent with responses to the baseline questionnaire.

### 3.7 Baseline CD4 count

The distributions of baseline CD4 cell counts (based on the day of enrolment) were very similar in each country (see Table 4). The median CD4 count at study enrolment was 272 (IQR: 124‐522) in Kenya and 294 (IQR: 135, 464) in South Africa. More than a third of patients presented for HIV care with advanced disease based on a CD4 count criterion alone (CD4 < 200 cells/mm^3^) in Kenya and South Africa respectively. Very advanced disease (CD4 < 100 cells/mm^3^) was also common, with 21% in Kenya and 18% in South Africa presenting with very advanced disease.

**Table 4:** Baseline CD4 cell counts and stratified by self‐reported symptoms and prior treatment status^a^

Baseline CD4 count	Kenya (SLATE I)			South Africa (SLATE II)		
	(N = 221)			(N = 273)		
Median (IQR)	272 (124, 522)			294 (135, 464)		
	Total	0 symptom	≥1 symptoms	Total	0 symptom	≥1 symptoms
<100 cells/mm^3^	21% 46	6% 14	14% 32	18% 48	4% 10	14% 38
≥100 and <200 cells/mm^3^	16% 36	10% 22	7% 14	18% 51	8% 22	11% 29
≥200 cells/mm^3^	63% 139	43% 95	20% 44	64% 174	41% 111	23% 63
Number treatment naïve	206			242		
Median (IQR) if treatment naive	278 (133,525)			291 (136,464)		
Number prior default	15			31		
Median (IQR) if prior default	195 (64, 408)			346 (128, 449)		

a The percentages reported in Table 4 are based on the total for each country. Underneath the percentage is the number for each.

While most patients presenting with advanced disease reported one or more of the symptoms listed in Table 3, but 16% of all patients in Kenya and 12% of all patients in South Africa presented with advanced disease and were asymptomatic. Roughly 1 in 20 patients (6% in Kenya, 4% in South Africa) presented with CD4 counts <100 cells/mm^3^ and were asymptomatic.

Two‐thirds of all patients presented higher CD4 counts ≥200 cells/mm^3^. While most of these patients were asymptomatic, one in five patients had higher CD4 cell counts (>200 cells/mm^3^) but reported at least one symptom listed in Table 3.

In Kenya, 15 of the 221 patients in Table 4 reported as prior defaulting, and these prior defaulters had a somewhat lower CD4 cell counts than those reporting to be treatment naïve. In South Africa, however 31 of the 273 patients in Table 4 reported as prior defaulting, and these prior defaulters had somewhat higher CD4 cell counts than for those reporting to be treatment naïve.

The two study populations described here, both presenting for HIV care and treatment, share six characteristics. First, more than half of patients presenting during mid‐2017 to mid‐2018 to facilities in both countries (62% in Kenya and 55% in South Africa) stated that they had not previously tested positive for HIV. This includes patients who had never tested as well as those testing negative previously. While these numbers may be somewhat inflated, with some patients preferring not to reveal a previous positive test, they remain worrisome. Efforts to expand testing—and to facilitate immediate treatment initiation for all those testing positive and to reinforce HIV prevention for those testing negative—remain critical for the success of universal treatment.

Second, generally all patients stated that they preferred to initiate ART on that same day if recommended by the clinician. A stated short‐term preference does not, of course, ensure long‐term adherence, but this result from 98% of patients in both countries does suggest that once patients learn their infected status, they are eager to move forward with treatment.

Third, about 1 in 10 patients were returning to the clinic for treatment after prior defaulting. In Kenya, these prior defaulters presented with substantially lower CD4 cell counts than did naïve patients, while in South Africa the CD cell count distribution was similar in both groups. As an increasing proportion of patients re‐enter treatment after dropping out sometime in the past 10, 11, understanding reasons for prior default and developing procedures for overcoming previous barriers to retention will also grow in importance.

Fourth, a large proportion of patients presenting at public sector clinics in the two study countries report symptoms that can be indicative of tuberculosis, cryptococcal meningitis, and/or other serious conditions (40% in Kenya, 47% South Africa), with symptoms of tuberculosis most common. Multiple symptoms were common in Kenya (27% with 3 or more symptoms), but less so in South Africa (8% with 3 or more symptoms).

Fifth, more than a third (36‐37%) of patients presenting from mid‐2017 to mid‐2018 had advanced HIV disease (CD4 ≤ 200) at presentation, and a fifth had very advanced disease (CD4 < 100). These proportions remain similar to those estimated before implementation of UTT 12. For example, in Nairobi, 32.9% of a cohort presenting for HIV care between April 2013 to June 2015 had advanced HIV disease (<200 or WHO Stage IV) 13. In South Africa during 2016, 32.9% of patients presenting for care had a CD4 cell count <200 cells/mm^3^ 14. We acknowledge that the populations accessing care before and after UTT population are not exactly comparable, because some patients in the “before” population who believed themselves to have high CD4 counts, and thus ineligible for ART, may not have sought clinical care. That said, advanced disease at presentation remains an important challenge, with costs in terms of patient prognosis, HIV transmission risk and healthcare expenditure 15.

And finally, a small but important number of patients presented with advanced HIV disease (CD4 < 200 mm^3^) and were asymptomatic. A lack of a consistent correlation between CD4 cell count and reported symptoms is consistent with prior literature 10. Current guidelines suggest that in the absence of immediate point‐of‐care CD4 count results, asymptomatic patients can be initiated on ART, with adjustments made or additional tests and/or services provided as soon as the CD results are available. In the standard of care arms in our Kenya and South Africa trials, which are not discussed here, we observed a range of clinician behaviour with regard to ART initiation among symptomatic patients. In some sites, clinicians adhered strictly to guidelines and deferred ART initiation if any relevant symptoms were reported (symptoms listed in Table 3); while in other sites, guidelines were adhered to less strictly.

This analysis has important limitations. First, while the study sites were all typical primary healthcare clinics in South Africa and typical hospital‐based HIV clinics in Kenya, they were geographically clustered in each country, making generalizability to the rest of the country uncertain. Second, by necessity, we excluded prior to randomization patients who were not physically or emotionally able to participate, leaving us with a potentially healthier sample than the overall population. And third, it is possible that patients who declined to participate were systematically different from study participants.

## 4 Conclusions

As long as a substantial proportion of patients continue to present with either low CD4 counts or symptoms of illness, clinical staff will continue to make decisions about eligibility for ART initiation (despite the presence of relevant symptoms) and about the need for additional care (despite the absence of relevant symptoms). While healthcare providers around the world already face these decisions on a daily basis, a goal of future research should be to provide better guidance to the nurses, clinical officers, and in some cases lay health workers, to maximize immediate ART initiation and identify and respond to patients who require additional care.

## Competing interests

WDFV sits on antiretroviral initiation guideline committees, both local and international, has accepted speaking honoraria from multiple manufacturers of antiretrovirals and is on several advisory boards. The remaining authors declare that they have no competing interests.

## Authors’ contributions

SR conceived of and designed the work. ATB, MM, BAL, IT, MB and WDFV contributed to designing the work. ATB, MM, BAL and SR wrote the first draft of the manuscript. All authors reviewed and revised the manuscript, approved the manuscript's results and conclusions, and have read, and confirmed that they meet ICMJE criteria for authorship.
